# Extracorporeal circulation in theory and practice

**DOI:** 10.1051/ject/2023011

**Published:** 2023-06-28

**Authors:** Alfred H. Stammers

**Affiliations:** 1 Vice President of Clinical Quality and Outcomes Research, Medical Department, Specialty Care 3 Maryland Farms, Suite 200 Brentwood Tennessee 37027 USA


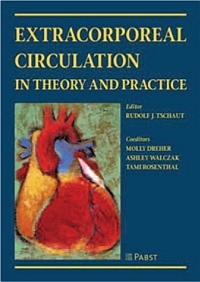


As we approach nearly 70 years since the first successful use of the heart-lung machine to provide a source of extracorporeal circulation so that surgeons could repair a congenital heart defect in a young lady in Philadelphia, Pennsylvania, USA, we marvel at the tremendous impact this technological wonder has had on saving millions of people throughout the world. Progress in perfusion practice rapidly advanced throughout the 1960’s and 1970’s, as manufacturers of cardiopulmonary products developed and refined apparatus used in the conduct of cardiopulmonary bypass with a focus on safety and reproducibility. In the 30 years that followed there was an intense research focus directed at understanding the negative pathophysiological consequences of extracorporeal flow. Concurrently efforts evolved to reduce extracorporeal induced hyperinflammation with efforts directed at improving the biocompatibility of foreign surfaces, reducing air-to-blood interactions, and by gaining a better understanding of the body’s complex response to inflammation. While the pursuit of ameliorating the consequences of blood flow across non-endothelialized surfaces continues, and results in hundreds of yearly publications in surgical, anesthesia, and perfusion journals, major texts on extracorporeal circulation occur less frequently. That is why the recent publication of *Extracorporeal Circulation in Theory and Practice*, by the editors Tschaut, Dreher, Walczak and Rosenthal, is received as a welcome addition to the science of perfusion.

The 729 page inclusive monograph consists of 18 sections with 61 chapters that address a myriad of subject matter. While the majority of authors are from the United States many are also from Europe, primarily from Germany, as well as Australia. The historical chapter on perfusion focuses primarily on extracorporeal circulation and is well-illustrated containing citations not normally found in other reviews. The chapter on the interactions of blood with artificial surfaces serves as a good introduction to the challenges of flow across non-endothelialized surfaces. The chapters on hemodynamics and monitoring both contain succinct reviews of the physio-hydraulic principles of fluid movement and will aid the entering perfusionists in grasping these principles.

The most useful information on extracorporeal circulation begins in Section 5 and focuses on the mechanical and physical processes associated with cardiopulmonary bypass. The chapter on extracorporeal surfaces was especially well-written and summarized issues related to hemocompatibility with an efficient segue to the ensuing chapter on surface coatings. Section 7 is well constructed containing a comprehensive evaluation of the principles of extracorporeal circulation with the only redundancy occurring in the two chapters dealing with cannulation. The pathophysiological consequences of extracorporeal circulation on various organ systems is nicely reviewed and well-referenced in Section 9. Extracorporeal blood management is addressed in Sections 10 and 11 and emphasizes the many modalities that are integral to perfusion and hemodialysis, and the associated evidence to support, or refute, inherent benefits of these technologies. The chapters on long-term mechanical support were informative. It was refreshing to see an entire Section (13) devoted to congenital heart disease and perfusion with emphasis on cerebral monitoring. A hodgepodge of special perfusion techniques are found in Section 14 and include extracorporeal circulation during pregnancy which is primarily focused on perfusion with a brief mention of concerns during extracorporeal life support (ECLS). The chapter on accidental hypothermia was particularly well-written and contained important considerations when managing these challenging cases.

It seemed a more logical placement of the chapters on ECLS would have been in Section 12 where long-term mechanical support was reviewed. Nevertheless the Editors are to be commended for devoting a significant portion of the text to ECLS which has undergone significant expansion in the past two decades, especially in the adult population, resulting in vast technological improvements which have enhanced outcomes in these severely ill patients.

While it understandable that the editors wished to produce an inclusive treatise on all aspects of extracorporeal circulation, there are a number of chapters that could have been omitted (anatomy and physiology, and blood) since most entering-level practitioners receive in-depth exposure to this subject matter in other course work. Also there was no mention of accompanying perfusion techniques in the coronary artery bypass grafting and heart valve surgery chapters, which would have improved them. While the chapter on tissue engineering and regenerative medicine was inclusive and extremely well-referenced, it seems out of place in this textbook. Another minor issue concerns the references found in most chapters. While it is understandable that text books contain many historical references, the citations in many of the chapters were quite old and should have been updated with more recent work. Also, there is a lack of standardization in how references are cited throughout the text with a number of chapters following the APA format and others using the Vancouver style.

Overall *Extracorporeal Circulation in Theory and Practice* will serve a useful purpose in educating individuals at the start of their training, as well as supplementing the knowledge to both perfusionists and other health professionals involved in the practice of cardiopulmonary bypass and associated technologies.

Reviewed by Alfred H. Stammers, MSA, CCP (Emeritus)

Vice President of Clinical Quality and Outcomes Research, Medical Department, Specialty Care, 3 Maryland Farms, Suite 200, Brentwood, Tennessee 37027, USA

Email: astammers@aol.com

